# Phosphorylation of ΔNp63α via a Novel TGFβ/ALK5 Signaling Mechanism Mediates the Anti-Clonogenic Effects of TGFβ

**DOI:** 10.1371/journal.pone.0050066

**Published:** 2012-11-16

**Authors:** Pratima Cherukuri, Andrew J. DeCastro, Amanda L. Balboni, Sondra L. Downey, Jennifer Y. Liu, Justine A. Hutchinson, James DiRenzo

**Affiliations:** 1 Department of Pharmacology and Toxicology, The Audrey and Theodor Geisel School of Medicine at Dartmouth, Hanover, New Hampshire, United States of America; 2 Program in Experimental Molecular Medicine, The Audrey and Theodor Geisel School of Medicine at Dartmouth, Hanover, New Hampshire, United States of America; 3 Department of Biological Sciences, Dartmouth College, Hanover, New Hampshire, United States of America; University of Pittsburgh, United States of America

## Abstract

Genetic analysis of *TP63* implicates ΔNp63 isoforms in preservation of replicative capacity and cellular lifespan within adult stem cells. ΔNp63α is also an oncogene and survival factor that mediates therapeutic resistance in squamous carcinomas. These diverse activities are the result of genetic and functional interactions between TP63 and an array of morphogenic and morphostatic signals that govern tissue and tumor stasis, mitotic polarity, and cell fate; however the cellular signals that account for specific functions of *TP63* are incompletely understood. To address this we sought to identify signaling pathways that regulate expression, stability or activity of ΔNp63α. An siRNA-based screen of the human kinome identified the Type 1 TGFβ receptor, ALK5, as the kinase required for phosphorylation of ΔNp63α at Serine 66/68 (S66/68). This activity is TGFβ-dependent and sensitive to either ALK5-directed siRNA or the ALK5 kinase inhibitor A83-01. Mechanistic studies support a model in which ALK5 is proteolytically cleaved at the internal juxtamembrane region resulting in the translocation of the C-terminal ALK5-intracellular kinase domain (ALK5^IKD^). In this study, we demonstrate that ALK5-mediated phosphorylation of ΔNp63α is required for the anti-clonogenic effects of TGFΒ and ectopic expression of ALK5^IKD^ mimics these effects. Finally, we present evidence that ultraviolet irradiation-mediated phosphorylation of ΔNp63α is sensitive to ALK5 inhibitors. These findings identify a non-canonical TGFβ-signaling pathway that mediates the anti-clonogenic effects of TGFβ and the effects of cellular stress via ΔNp63α phosphorylation.

## Introduction

TP63 is a member of the p53 family of transcriptional regulators [1] that preserves long-term regenerative stasis in diverse epithelial structures by maintaining the replicative capacity of adult stem cells [2], [3]. Several lines of evidence also implicate TP63 in multiple aspects of cancer initiation and progression. The mechanisms by which TP63 carries out these critical functions in development and disease are not fully understood, and progress toward this end is complicated by the fact that TP63 encodes as many as eight p63 isoforms. Differential usage of distal and proximal promoters results in isoforms with (TAp63) or without (ΔNp63) an amino-terminal trans-activation domain homologous to that of p53. Additionally alternative mRNA splicing results in C-terminal diversity. ΔNp63α is the predominant TP63 isoform in regenerative compartments of diverse epithelial structures and tumors of squamous epithelial origin. Isoform specific knockouts unambiguously indicate that the ΔNp63 isoforms account for the maintenance of replicative capacity [4], [5]. A second layer of complexity arises from studies indicating that ΔNp63α occupies greater than 5000 sites across the human genome and that these sites correlate with activation and repression of transcriptional targets [6]. Finally, the stability, transcriptional activity and cellular localization of TP63 gene products are regulated post-translationally by multiple phosphorylation events as well as by ubiquitination [7], SUMOylation [8] and ISGylation [9]. This combination of isoform diversity, widespread genomic occupancy, and post-translational regulation underscores the challenges of identifying the regulatory mechanisms and transcriptional targets of TP63 that account for its complex role in tissue and tumor stasis.

ΔNp63α has been shown to play important roles in cancer initiation and progression suggesting that pharmacologic strategies that disrupt the activity of ΔNp63α have the potential for therapeutic benefit. ΔNp63α is an oncogene that suppresses the activity of the Ink^4A^/ARF locus [10] and opposes the tumor suppressive effects of cellular senescence [11], [12] suggesting a role in oncogenic initiation [13]. TP63 is amplified at the genomic level in 9.7% of head and neck squamous cell carcinomas, 12.9% of serous ovarian carcinomas, 23% of squamous cervical carcinomas and 28.5% of lung squamous cell carcinomas (http://cbioportal.org) [14]. Presently the relationship between this amplification and cancer initiation is unknown, however ΔNp63α is a survival factor that opposes a pro-apoptotic gene expression program [15], [16] suggesting a correlation between TP63 amplification and therapeutic resistance. Other studies implicate ΔNp63α in cellular quiescence [17], which may account for the broad-spectrum resistance of squamous carcinomas [17], which may account for the broad-spectrum resistance to cytotoxic therapeutics. These studies implicate ΔNp63α in a diverse array of processes associated with cancer initiation and progression and this highlights the need to identify cellular signals governing these diverse activities.

TGFβ is a highly pleiotropic cytokine that governs diverse aspects of cell biology including cell cycle progression, senescence, differentiation and apoptosis [18], [19]. Its effects are mediated through a heterotetrameric TGFβ receptor complex consisting of two molecules of the Type I TGFβ receptor (ALK5) and two molecules of the Type 2 TGFβ Receptor (TGFβR2) [20], [21]. Both ALK5 and TGFβR2 possess intrinsic serine/threonine kinase activity, and upon ligand binding, TGFβR2 trans-phosphorylates ALK5 and enhances ALK5 kinase activity. This results in ALK5-mediated phosphorylation and nuclear translocation of the canonical effectors of TGFβ signaling, SMAD2 and SMAD3 [18]. In addition to this canonical TGFβ signaling pathway, the TGFβ receptor complex converges on several other cellular signaling pathways [22], [23]. Recently, ChIP-Seq studies indicate that SMAD3 co-occupies genomic loci with transcription factors that are master regulators of diverse cell types including Oct4 in ES cells, MyoD1 in myotubes and Pu.1 in the B-cell lineage [24] and may explain the cellular context-specific effects of TGFβ. TGFβ is also known for its regulation of postnatal mammary gland development and for its prominent role to act as a tumor suppressor by preventing mammary epithelial cell proliferation [25]. These studies implicate multiple signal transduction pathways in the pleiotropic effects of TGFβ.

The involvement of ΔNp63α in cancer initiation and progression suggests that signaling pathways that govern ΔNp63α activity or stability may be targeted for therapeutic benefit. Multiple phosphorylation sites have been identified within ΔNp63α and other TP63 isoforms [26]–[29] however, the underlying signaling pathways and functional consequences are known for only a subset of these modifications. In response to cisplatin, ΔNp63α is phosphorylated by c-Abl and this is required for cell viability [30]. In response to DNA damage, HIPK2 phosphorylates ΔNp63α and promotes its degradation [31]. Additionally, Serine 66/68 (S66/68) of ΔNp63α are phosphorylated in response to ultraviolet irradiation [29]. A recent report indicates that this phosphorylation event is associated with the elaboration of progenitors from stem cells in the skin [28]. These two observations suggest that phosphorylation at S66/68 might mediate a mitogenic response to cellular stress in which stem cells respond by dividing to produce new progenitors. To identify the cellular signaling pathways that promote S66/68 phosphorylation, an siRNA-based screen of the human kinome was conducted. The Type 1 TGFβ Receptor, ALK5, was identified as a kinase that is necessary for phosphorylation of ΔNp63α at S66/68. Consistent with this finding, TGFβ was sufficient to cause phosphorylation of ΔNp63α, and selective inhibitors of the ALK5 kinase blocked this phosphorylation. Mechanistic studies support a model in which TGFβ stimulation initiates the proteolytic cleavage of ALK5 within the juxtamembrane resulting in the nuclear translocation of ALK5^IKD^. This translocation results in the phosphorylation of ΔNp63α, which is required for the anti-clonogenic activities of TGFβ. Consistent with this model, ectopic expression of ALK5^IKD^ is sufficient to phosphorylate ΔNp63α and recapitulates the anti-clonogenic and anti-proliferative effects of TGFβ. Finally, we show that phosphorylation of ΔNp63α at S66/68 in response to ultraviolet (UV) irradiation is mediated by ALK5 indicating that the ALK5/ΔNp63α signaling pathway may mediate aspects of the cellular response to stress. Together these studies identify ΔNp63α as a target of a novel non-canonical ALK5 signaling pathway that mediates cellular responses to TGFβ.

## Materials and Methods

### Cell Culture and Treatments

Immortalized Mammary Epithelial Cells (IMECs) [32] were cultured in MEGM complete media (Lonza CC-3051) with 50 ug/ml puromycin and Penicillin/Streptomycin. Treatments with TGFβ1 and A83-01 were performed in basal MEGM media with 50 ug/ml puromycin, 0.1%BSA and Penicillin/Streptomycin on 20% confluent cells plated the night before. H1299s (ATCC# CRL-5803) were cultured in Dulbecco’s Modified Eagle Medium (DMEM) supplemented with 10% FBS and Penicillin/Streptomycin. All TGFβ1 and A83-01 treatments of H1299 cells were performed in DMEM media supplemented with 0.1% BSA and Penicillin/Streptomycin. Recombinant TGFβ1 (R&D systems) was used at a 500 pM concentration for 1 hr or as indicated. The ALK5 kinase inhibitor, A83-01 (Tocris), was used at a concentration of 2 uM for 1 hr prior to TGFβ1 treatment, or as indicated. For UV experiments, cells plated at 30% confluence were treated with UV radiation at 50 J/m^2^ and lysates were harvested 1 hr later. For stability experiments, cells were plated at 30% confluency and treated with 20 ug/ml cycloheximide (CHX).

### Plasmids and Transfections

The expression construct pcDNA3.1-ΔNp63α-WT was generated as previously described [33]. pcDNA3.1- ΔNp63α-AA (phospho ablative mutant) was made via the conversion of the amino acid serine to alanine (SSAA) at positions 66 and 68 of the DNA binding domain of TP63 using the Statagene Quik Change II site-directed mutagenesis kit (Cat# 200523-5) with the following set of primers;

sense primer 5′-CGATGCTCTCGCTCCAGCACCCGCCATCCCCTCC-3′, and antisense primer 5′-GGAGGGGATGGCGGGTGCTGGAGCGAGAGCATCG-3′. TGFβRI expression constructs- pRK5 TGFβRI wt Flag, pRK5 TGFβRI (T202D) Flag, and pCMV5B- TGFβRI (K232R) were purchased from Addgene (Cambridge MA) and were used as previously described [34]. pRK5- TGFβRII Flag expression vector [21] was purchased from Addgene (Cambridge, MA). The TGFβRI-GFP plasmid was a kind gift from Dr. J. C. Zwaagstra (McGill University, Canada). pcDNA3.1- TGFβRI^IKD^ was generated by PCR amplification of the kinase domain region of the pRK5 TGFβR1-wt-Flag plasmid using the following primers designed to incorporate Nhe1 and HindIII overhangs; sense primer, 5′-GATCGCTAGCATGATTGTGCTACAAGAAAGCATC-3′ and the antisense primer 5′-GATCAAGCTTCTTGTCGTCGTCGTCCTTGTAGTC-3′. Fragments generated from PCR were then cloned into the pcDNA3.1 vector. All plasmid transfections were carried out using Lipofectamine2000 (Invitrogen, Cat#11668) according to manufacturers protocol.

### Kinome Screen

The Silencer Select Human Kinase siRNA Library (Catalog#4397918) from Ambion was used to perform a genome wide kinome screen. The assay was performed in a 96 well format. Three different siRNAs for a single gene were co-transfected per well at a concentration of 0.5 pmoles using siPORT NeoFX Transfection Agent (Cat#AM4511). Forty-eight hours post siRNA transfection; cells were infected with ΔNp63α-WT adenovirus resulting in 90% infection efficiency. Quantitative immunofluorescence assays were then performed using the anti-phospho-p63 (Ser160/162) antibody. Immunofluorescence assays were performed as described below. Fluorescent values were obtained using the Molecular Devices Gemini XS Fluorescent Microplate Reader (EX: 540, EM: 570 and Cutoff: 570). Raw fluorescence values were normalized against total cell count, three different negative controls and a positive control. To obtain total cell count, cells after IF readings were stained with crystal violet. After a rigorous wash with distilled water, stain from the cells were eluted in a 20% methanol and 10% acetic acid solution, values were read at 595 nm using the Molecular Devices Thermo Max Microplate reader at 595 nm.

### Small-interfering RNA (siRNA) Transfection

Silencer select pre validated small-interfering RNA against TGFβRI and TGFβRII (Catalog #: 4390824) were purchased from Ambion. Ambion’s siPORT NeoFX Transfection Agent was used in all siRNA transfections according to the manufacturer’s protocol. Ambion Silencer®Negative Control #2 siRNA (Cat#AM4613) was used as control for all siRNA transfections. Forty eight hours post siRNA transfection, cells were transfected with GFP, ΔNp63α-WT or ΔNp63α-AA mutant plasmids, and harvested 24 hrs after plasmid addition in 1XSDS lysis buffer for further analysis by immunoblotting.

### Western Analysis and Immunofluorescence

Cells were lysed in 1XSDS lysis buffer with β-mercaptoethanol and were resolved by 10% SDS- PAGE. Antibodies used for western and Immunofluorescence were anti-p63 Clone 4A4 (Sigma, Cat#P3737) for total P63, anti-phospho-p63 (Ser160/162) (Cell signaling, Cat#4981), anti-phospho-Smad2 (Ser465/467) (Cell signaling, Cat#3108), anti-Smad2 (Cell signaling, Cat#3122), anti-TGFβ RII (L-21) (Santa Cruz, Cat#sc-400), anti-TGFβ RI (V-22) antibody from (Santa Cruz (, Cat#sc-398) and anti-β-Actin antibody from (Cell signaling (, Cat#3700). All primary antibodies were detected with their respective secondary HRP- conjugated antibodies using Millipore chemiluminescence. Primary antibodies for Immunofluorescence assays were detected with either anti-mouse-Alexa Fluor 488 (Invitrogen#A11029) or anti-rabbit-Alexa Fluor 555 (Invitrogen#A21429).

### Colony Formation Assay

IMEC cells were plated at 1000 cells per well in a 6-well dish. Cells were fed every other day with TGFβ1± A83-01 for 10–14 days. Cells transfected with p63 constructs were selected for in the presence of G418. Colonies were fixed in ice-cold 80% methanol and stained with 0.5% crystal violet.

### In vitro Kinase Assay

Recombinant ALK5 kinase was produced by transfecting H1299 cells with an expression vector programmed to produce C-terminally flag-tagged ALK5 Receptor. Immunoprecipitation of the Flag-tagged ALK5 kinase was performed using Peirce Classic IP kit and monoclonal ANTI-FLAG M2 antibody(Sigma Cat # F1804). Recombinant ALK5 was eluted from beads by incubation with 100 ug/ml of soluble flag peptide (Sigma Cat # F3290) in 10 mMTris, 150 mM NaCl2, pH7.4 buffer. GST-ΔNp63α was produced via IPTG induced expression in Y1090 cells. Bacteria were collected by centrifugation and lysed by sonication in the presence of a cocktail of protease inhibitors. Lysates were cleared by centrifugation and GST-ΔNp63α was enriched on glutathione-sepaharose beads. For the kinase reaction either 1 or 5 µL of soluble ALK5 was added to fixed amounts of GST-ΔNp63α bound to beads in 1X kinase buffer (50 mM HEPES, 5 mM Mgcl2,1 mM Cacl2) with 10 uM ATP. Reactions were incubated at 30°C for 30 min. Reactions were stopped by the addition of 2 × SDS Sample Buffer and western blots were done as described previously.

### Aldefluor Assay

ALDH^high^ and ALDH^low^ populations in IMEC cells were identified using the Aldefluor assay kit (Stem Cell Technologies). IMEC cells were plated at 25% confluence and treated with Vehicle, TGFβ1 or A83-01 for 24 and 48 hours. Cells were then harvested and stained with ALDEFLOUR reagent as per the manufacturers protocol.

### Cell Cycle Analysis and Subcellular Fractionation

Cells were collected by trypsinization and gentle centrifugation before being re-suspended in ice cold PBS. An equal volume of ice cold 80% methanol was added with gentle vortexing and cells were fixed on ice for 30 minutes. Fixed cells were collected by centrifugation and re-suspended in PBS supplemented with 0.5 µg/ml of RNAse A. After 45 minutes at 37°C, cells were stained with propidium iodide and samples were analyzed on a BD FACScan instrument. Nuclear and cytoplasmic extracts were prepared using the EpiQuikTM Nuclear Extraction Kit I according to the manufacturer’s protocol.

## Results

### A Small Interfering RNA Screen of the Human Kinome Identifies ALK5 as a Putative ΔNp63α Kinase

**Figure 1:**
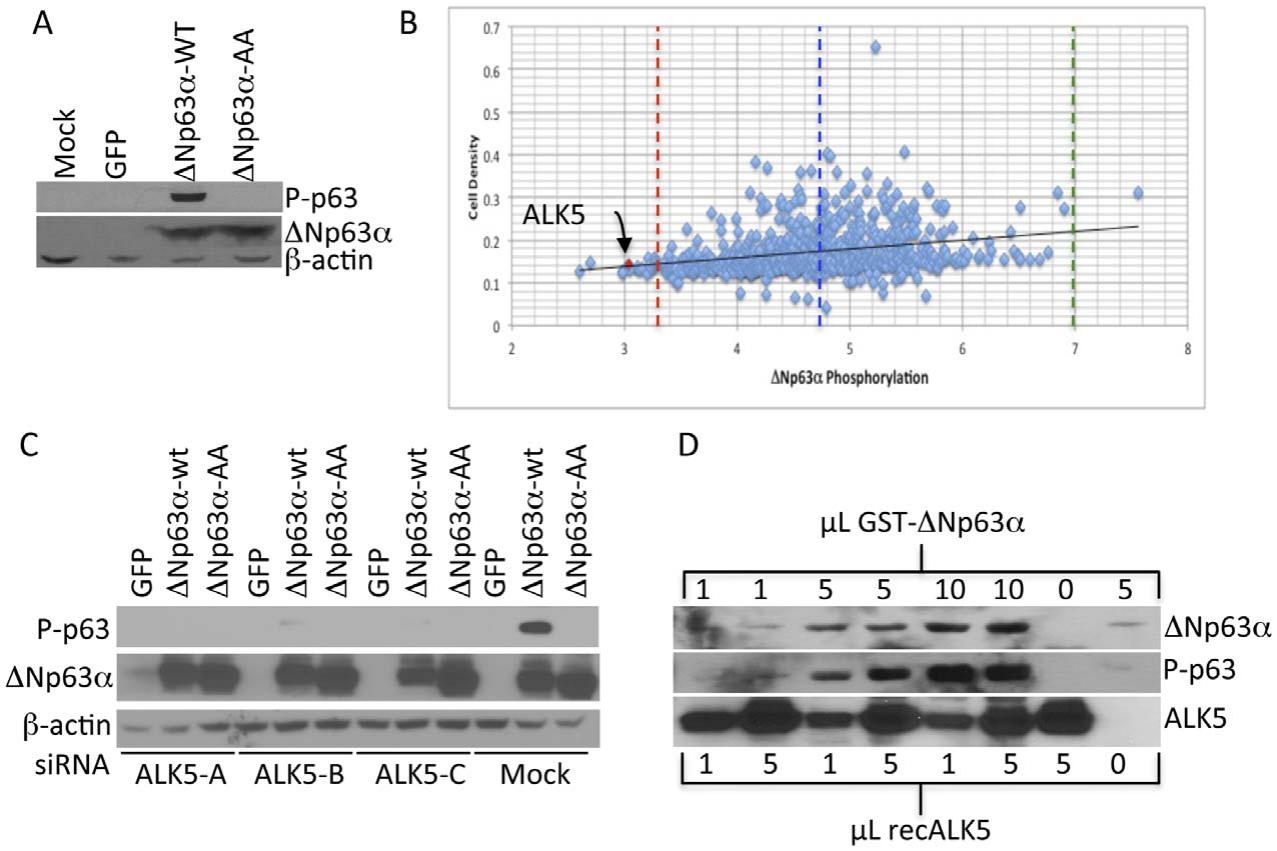
An siRNA-based screen of the human kinome identifies ALK5 as a putative ΔNp63α kinase. **A.** ΔNp63α-WT and phospho-ablative p63 mutant (ΔNp63α-AA) were transiently transfected into H1299 cells. Cell lysates were harvested after 24 hrs and the levels of phospho-P63, total p63 and β-actin were analyzed. **B.** Graphical representation of results of the phospho-P63 expression as indicated by normalized fluorescent values from the kinome screen. Each dot represents relative p-p63 abundance following treatment with siRNA (n = 3) directed against a single kinase. The green dashed line represents mean positive control value and the red dashed line represents the mean negative control. The blue dashed line represents the mean phospho-p63 score in the screen. All the hits below the red dotted line were considered as possible kinases responsible for phosphorylating ΔNp63α. **C.** Three different siRNAs targeted against ALK5 were transfected into H1299 cells, 48 hrs later cells were transfected with either GFP, ΔNp63α-WT or ΔNp63α-AA expression vectors, whole cell lysates harvested after 24 hrs were analyzed for phospho-P63, total p63, and β-actin. **D.** Recombinant ALK5 phosphorylates ΔNp63α in vitro. Purified GST tagged ΔNp63α protein was incubated with purified Flag-ALK5 kinase for 30 min at 30°c and reactions were analyzed for phospho-P63, totalP63, and Flag-Alk5.

To identify the signaling pathways governing the diverse activities of ΔNp63α a siRNA-based screen of the human kinome was carried out in H1299 lung adenocarcinoma cells (Figure S1). H1299 cells do not express ΔNp63α but rapidly phosphorylate ectopic wild-type ΔNp63α (ΔNp63α-WT) but not a mutant allele in which serines at positions 66 and 68 were changed to alanine (ΔNp63α-AA) (Figure 1A). Pools of three kinase-specific siRNAs were transfected into H1299 cells. At 48 hours post transfection cells were infected with an adenovirus programmed to express ΔNp63α. Twenty four hours post infection, phospho-p63 levels were measured by immunofluorescence and quantitated using a fluorescent plate reader. Following this analysis, cells were stained with crystal violet to record cell density. Analysis of phospho-ΔNp63α immunofluorescence intensity normalized to cellular density (Figure 1B) resulted in the identification of several kinases (Table in Figure S1) with normalized phospho-ΔNp63α scores lower than the negative control (red dashed line). Among these was the Type 1 TGFβ Receptor (ALK5) and transfection of each of the three individual ALK5-directed siRNAs was sufficient to suppress ΔNp63α phosphorylation at S66/68, indicating that ALK5 was necessary for ΔNp63α phosphorylation (Figure 1C). To determine if ALK5 was sufficient to phosphorylate ΔNp63α H1299 cells were transfected flag-tagged ALK5 and recombinant ALK5 was isolated by anti-flag affinity chromatography and eluted with soluble flag-peptide. One and five µL of the eluted fraction was incubated with increasing amounts of bacterially expressed glutathione-S-transferase-ΔNp63α fusion protein. Western analysis revealed that recombinant ALK5 was able to phosphorylate ΔNp63α in vitro (Figure 1D). Given that both ALK5 and GST-ΔNp63α were affinity purified, these data support the assertion that ALK5 directly phosphorylates ΔNp63α. This in turn raises questions regarding the mechanisms by which a membrane-bound kinase can phosphorylate a nuclear protein. Finally, H1299 cells were co-transfected with ΔNp63α and a series of ALK5 expression plasmids that express wild type or mutant ALK5. Phospho-p63 western analysis indicated that ectopic ALK5 resulted in phosphorylation of ΔNp63α. Remarkably, a threonine to aspartate mutation T202D (T198D in rat ALK5 NP_036907.2) that constitutively activates TGFβ signaling [34] caused an increase in SMAD2 phosphorylation but was unable to phosphorylate ΔNp63α. Additionally a lysine to arginine mutation K232R (K226R in rat ALK5 NP_036907.2) in rat ALK5 that fails to mediate canonical TGFβ signaling [35] and fails to phosphorylate SMAD2 but is able to efficiently phosphorylate ΔNp63α (Figure S2). These observations suggest that the intramolecular determinants of SMAD2 phosphorylation, and by extension canonical TGFβ-signaling, are distinct from those required for ΔNp63α phosphorylation. Together these studies demonstrate that ALK5 is necessary and sufficient to phosphorylate ΔNp63α and suggest that the molecular mechanisms by which ALK5 phosphorylates ΔNp63α may be distinct from those governing phosphorylation of SMAD2.

### TGFΒ Stimulates ALK5-mediated Phosphorylation of ΔNp63 α via TGFβR2

**Figure 2:**
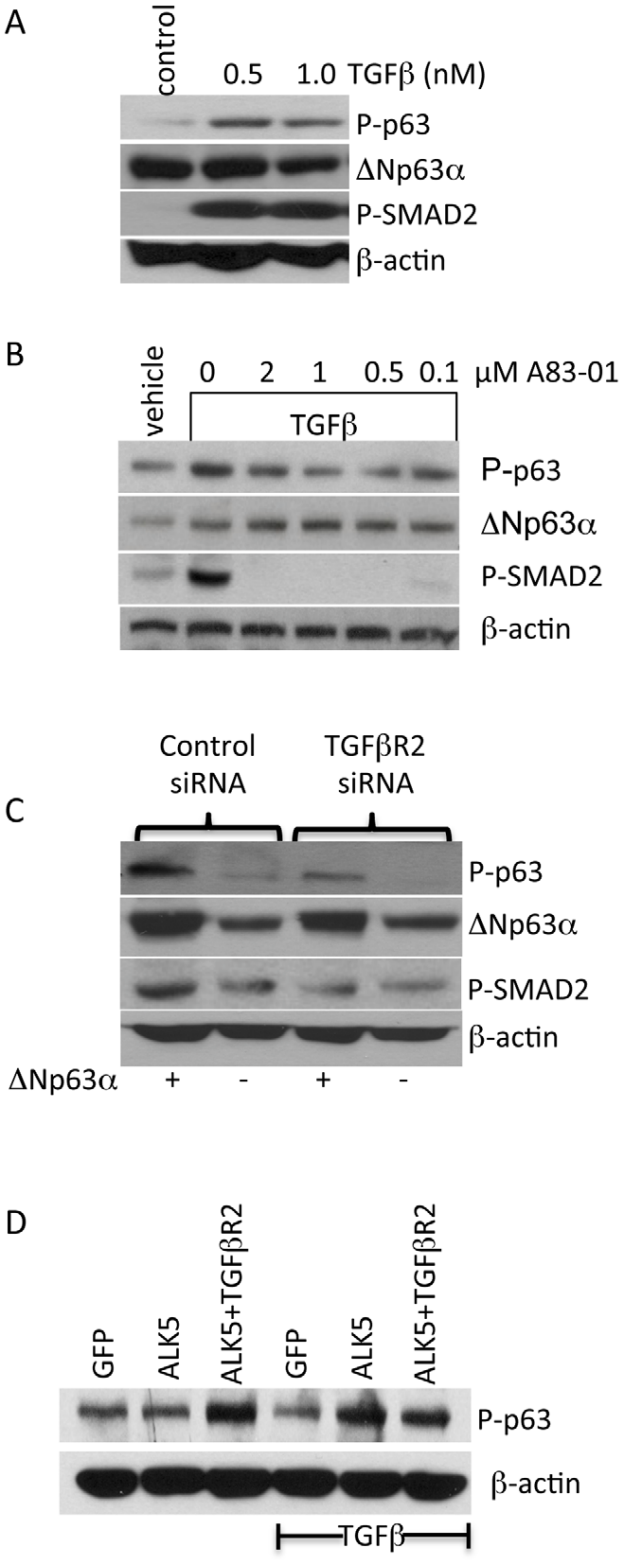
TGFβ stimulates of ALK5-mediated phosphorylation of ΔNp63α via TGFBR2. A. IMEC cells were treated with the indicated concentrations of TGFβ1 ligand for 1 hr. Whole cell lysates were harvested and analyzed by immunoblotting for levels of phospho-p63, total p63, phospho-SMAD2 and β-actin. **B.** ALK5-mediated phosphorylation of ΔNp63α is inhibited by A83-01**.** IMEC cells were treated with A83-01 at the indicated concentrations 1 hr prior to TGFβ1 treatment. Whole cell extracts were analyzed after 1 hr for phospho-p63, total-p63, phospho-SMAD2 and β-actin via immunoblotting. **C.** SiRNA targeted against TGFβR2 was transfected into IMEC cells, 48 hrs later cells were transfected with ΔNp63α expression vector or GPP control vector. Whole cell lysates were harvested 24 hrs later and analyzed for phospho-P63, total-P63, phospho-SMAD2 and β-actin. **D.** Ectopic expression of ALK5 and TGFβR2 is sufficient to phosphorylate ΔNp63α in a manner that is independent of TGFB.

The finding that ALK5 is necessary and sufficient for *Δ*Np63α phosphorylation suggested that TGFβ-signaling governs ΔNp63α phosphorylation. We therefore sought to determine if TGFβ stimulation was sufficient to enhance ΔNp63α phosphorylation. For these studies, an hTERT-immortalized mammary epithelial cell (IMEC) line was used due to its robust expression of ΔNp63α [32] and the fact that it is cultured in a chemically-defined media, which enables experimentation under TGFβ-depleted conditions. Results indicate that TGFβ stimulation increases phospho-ΔNp63α levels within one hour, indicating signaling kinetics similar to SMAD2 phosphorylation. This phosphorylation was inhibited by A83-01, a selective ALK5 kinase inhibitor [36] (Figure 2B), which supports the assertion that TGFβ mediated ΔNp63α phosphorylation requires ALK5 activity. This observation coupled to the fact that ALK5 possesses no inherent TGFΒ-binding capacity suggested the involvement of TGFβR2. SiRNAs that produce a substantial reduction in TGFβR2 expression (Figure S3) directed against TGFβ R2 were co-transfected into IMECs ± pcDNA-ΔNp63α. Phospho-p63 western analysis indicated that TGFβR2 is necessary for TGFβ-mediated phosphorylation of ΔNp63α and SMAD2 (Figure 2C). Under TGFβ-depleted culture conditions ectopic TGFβR2 promoted ALK5-mediated phosphorylation of ΔNp63α in a manner that was independent of TGFβ (Figure 2D, compare lanes 2 and 3). This result is consistent with studies indicating that TGFβ stimulates the physical association of TGFβR2 and ALK5 [37]. Together, these results indicate that TGFΒ initiates ALK5-mediated ΔNp63α phosphorylation via the canonical TGFβR2/ALK5 receptor complex.

### ALK5 Mediates Phosphorylation of ΔNp63α in Response to Ultraviolet Irradiation

**Figure 3:**
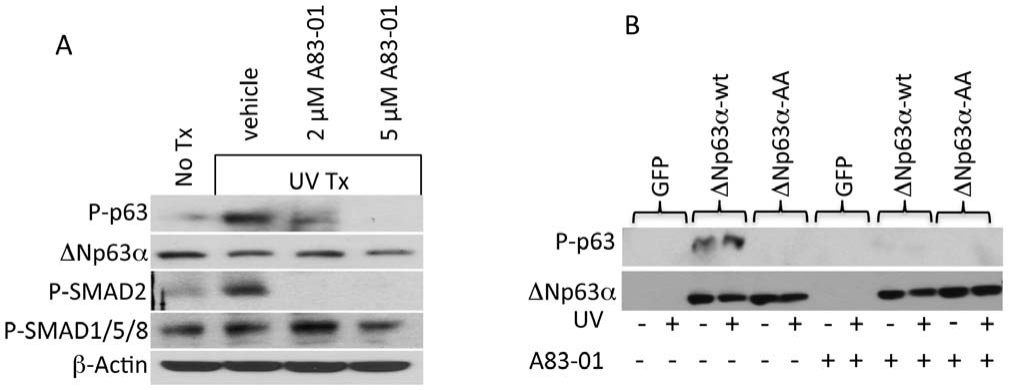
ALK5 mediates phosphorylation of ΔNp63α in response to ultraviolet irradiation. **A.** IMEC cells were plated and treated with the indicated amounts of A83-01 or vehicle for 12 hrs after which cells were exposed to 50 J/m^2^ UV radiation. Whole cell lysates were collected after 1 hr of UV treatment and analyzed by immunoblotting for phospho-P63, total-P63, phospho-SMAD2 and β-actin levels. Phospho-SMAD1/5/8 was used as a control to show that there were no off-target effects for A83-01 at the concentrations used. **B.** H1299 cells were transfected with GFP, ΔNp63α-WT or ΔNp63α-AA expression vectors and cells were treated with 2 µM A83-01 or vehicle control. Twenty-four hours later cells were exposed to 50 J/m^2^ UV radiation and whole cell extracts were collected after 1 hr to analyze the levels of phospho-P63, total-P63 and β-actin by immunoblotting.

Previous studies have shown that ΔNp63α is phosphorylated at S66/68 in response to ultraviolet (UV) irradiation [29]. Other studies have demonstrated increased TGFβ signaling in response to UV irradiation [38]–[40]. Additionally, recent studies have implicated TGFβ signaling in increased metastasis following ionizing radiation [41] and others have shown that ionizing radiation results in enhanced TGFβ signaling from the tumor microenvironment that results in pro-carcinogenic effects [42]. Together these studies indicate that ALK5 signaling mediates ΔNp63α phosphorylation in response to UV irradiation. To determine if ALK5 mediates UV-induced phosphorylation of endogenous ΔNp63α, IMECs were incubated in the presence or absence of A83-01 for 12 hrs and then subjected to UV irradiation. Western analysis indicated that UV irradiation stimulated phosphorylation of ΔNp63α was sensitive to A83-01, indicating that ALK5 was able to phosphorylate ΔNp63α in response to UV irradiation (Figure 3A). Interestingly, UV irradiation also increased phosphorylation of SMAD2 in a manner that was sensitive to A83-01 suggesting that the mechanism(s) by which UV irradiation induces TGFΒ-signaling most likely act upstream of ALK5. Similarly H1299 cells were transfected with GFP, ΔNp63α -WT or the phospho-ablative mutant, ΔNp63α-AA, and cells were either mock exposed or exposed to UV irradiation followed by treatment with A83-01 or vehicle. Western blot analysis indicated that phosphorylation of ΔNp63α at S66/68 increased in response to UV irradiation and that inhibition of ALK5 with A83-01 was sufficient to ablate ΔNp63α phosphorylation (Figure 3B). Together these studies indicate that ALK5 mediates UV-induced activation of TGFβ-signaling which leads to increased phosphorylation of SMAD2 and ΔNp63α. These studies implicate ALK5 in a previously unknown role in the cellular stress response and suggest that disruption of ΔNp63α phosphorylation may sensitize cells to diverse types of cellular stress. Coupled to studies indicating that ΔNp63α is a potent blockade to apoptosis in experimental models of HNSCC [16] and triple negative breast cancer [15] these studies identify a potential strategy to subvert ΔNp63α mediated drug resistance by inhibiting TGFΒ signaling.

### Nuclear Accumulation of the Intracellular Kinase Domain of ALK5 (ALK5^IKD^) in Response to TGFβ

**Figure 4:**
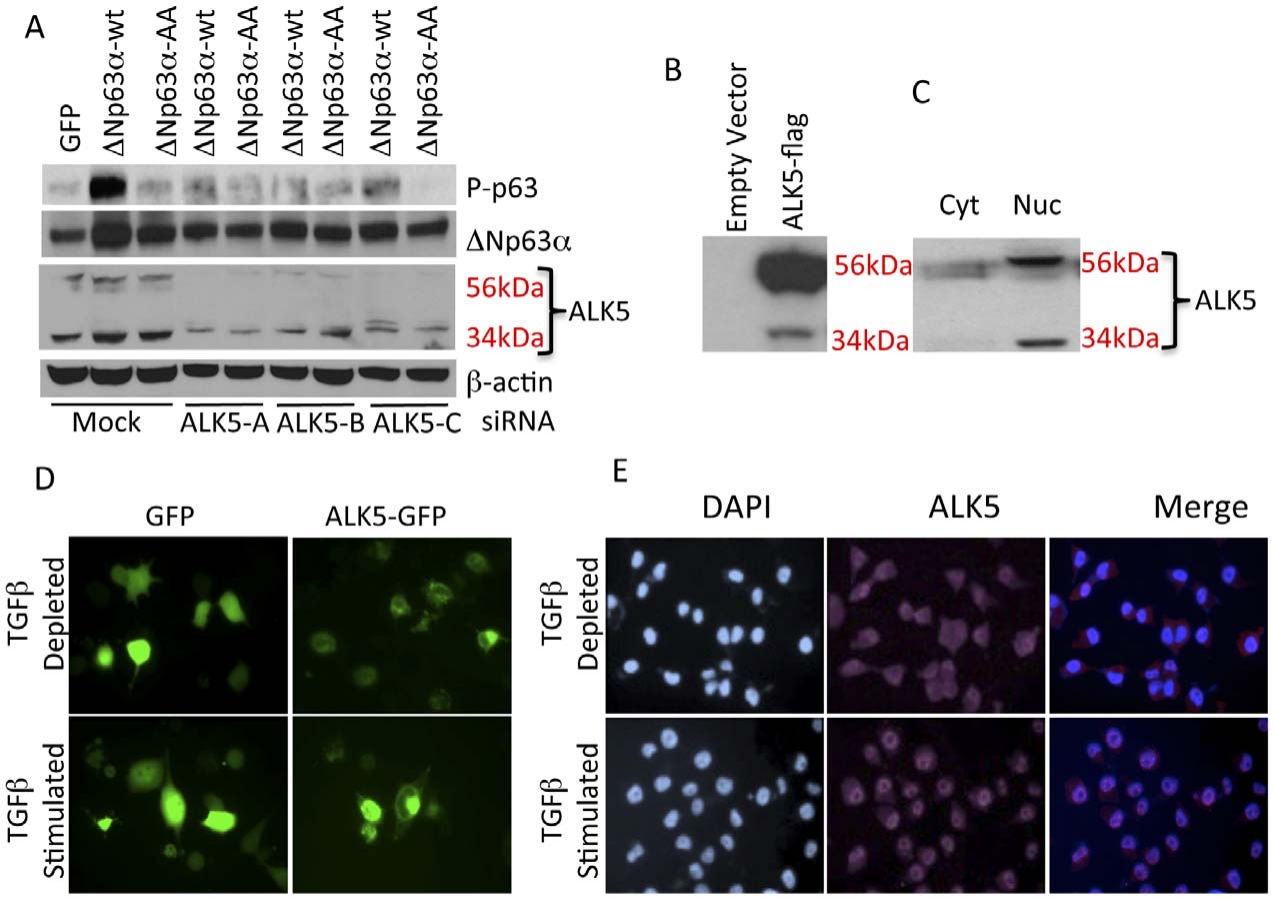
Nuclear accumulation of the intracellular kinase domain of ALK5 (ALK5^IKD^) in response to TGFβ. **A.** Three different siRNAs targeted against ALK5 were transfected into IMEC cells, 48 hrs later cells were transfected with GFP, ΔNp63α-WT and ΔNp63α-AA expression vectors, whole cell lysates were harvested after 24 hrs and analyzed for phospho-p63, total p63, TGFβR1 and β-actin. **B.** H1299 cells were transfected with an ALK5-WT-Flag expression vector, whole cell lysates were harvested and analyzed for full length and cleaved fragments with anti-Flag antibody. **C.** Analysis of ALK5 distribution in IMECs indicates that the 34 kDa C-terminal ALK5 fragment is present only in the nucleus. **D.** H1299 cells were transfected with ALK5-GFP expression vector, 6 hrs after transfection cells were treated with vehicle control or TGFβ1 for 8 hrs and imaged for subcellular distribution of GFP. **E.** H1299 cells were serum starved for 12 hr and then induced with vehicle control or TGFβ1 for 1hour. Cells were stained with anti- TGFβR1 (V-22) antibody. Depletion of TGFβR1in H1299cells by siRNA is shown as a control for the specificity of the antibody.

The identification of ALK5 as a ΔNp63α kinase and the demonstration that this event is initiated by TGFβ raised significant questions regarding the mechanisms by which a membrane bound kinase phosphorylates ΔNp63α, which is located in the nucleus. ALK5 activation is propagated through multiple transduction pathways, several of which rely upon diverse kinase activities [43]–[45]. To determine if any of these known pathways mediate ΔNp63α phosphorylation, phospho-ΔNp63α immunofluorescence data from the kinome-wide siRNA screen was re-evaluated. Results of this evaluation indicated that no other kinase known to be downstream of ALK5 was implicated in ΔNp63α phosphorylation (Figure S4). Additionally, western analysis of IMECs transfected with ALK5 siRNA identified a 34-kDa fragment that was sensitive to ALK5-directed siRNA and detectable with an antisera directed against the C-terminus of ALK5 (Figure 4A). The 34-kDa size coupled to selective detection with a C-terminally directed antibody suggested that this band is the product of proteolytic cleavage of ALK5 at or near the intracellular juxtamembrane region. To test this hypothesis, a plasmid encoding C-terminally-flag-tagged versions of wild-type ALK5 was transfected into H1299 cells and flag-tagged proteins were detected by western blot. Results indicated that ectopic expression of ALK5 results in a 56 kDa full-length receptor and a 34 kDa C-terminal fragment (Figure 4B) suggesting that exogenous ALK5 was processed in a manner that is similar or identical to endogenous ALK5. Since these experiments were done by transfecting cDNAs of wild-type and mutant ALK5, these results also demonstrate that the 34-kDa fragment is unlikely to be the result of alternative mRNA splicing. These observations support a model in which TGFβ stimulates proteolytic cleavage of ALK5 and that the 34 kDa ALK5 intracellular kinase domain (ALK5^IKD^) would preferentially localize in the nucleus. Western analysis of nuclear and cytoplasmic extracts from IMECs indicated that the 34-kDa ALK5 C-terminal fragment was selectively localized to the nucleus (Figure 4C). To determine if ALK5 is able to translocate to the nucleus, an ALK5-GFP fusion expression vector [46] was transfected into IMECs under TGFβ-depleted conditions and cells were then stimulated with vehicle or TGFβ. Fluorescence microscopy indicated that TGFβ stimulated the redistribution of ALK5-GFP the nucleus, consistent with the nuclear localization of the ALK5^IKD^ (Figure 4D). Similarly, stimulation of H1299 cells with TGFβ resulted in redistribution of ALK5 from the cytoplasm to the nucleus (Figure 4E). Transfection of ALK5-directed siRNA confirms the specificity of the immunofluorescent analysis (Figure S5). Together these observations support a mechanistic model in which TGFβ stimulation initiates the nuclear translocation of ALK5, thereby enabling phosphorylation of ΔNp63α.

### TGFβ is Anti-proliferative and Suppresses ALDH1 Activity and ΔNp63α Protein Levels in a Mammary Stem Cell Model

**Figure 5:**
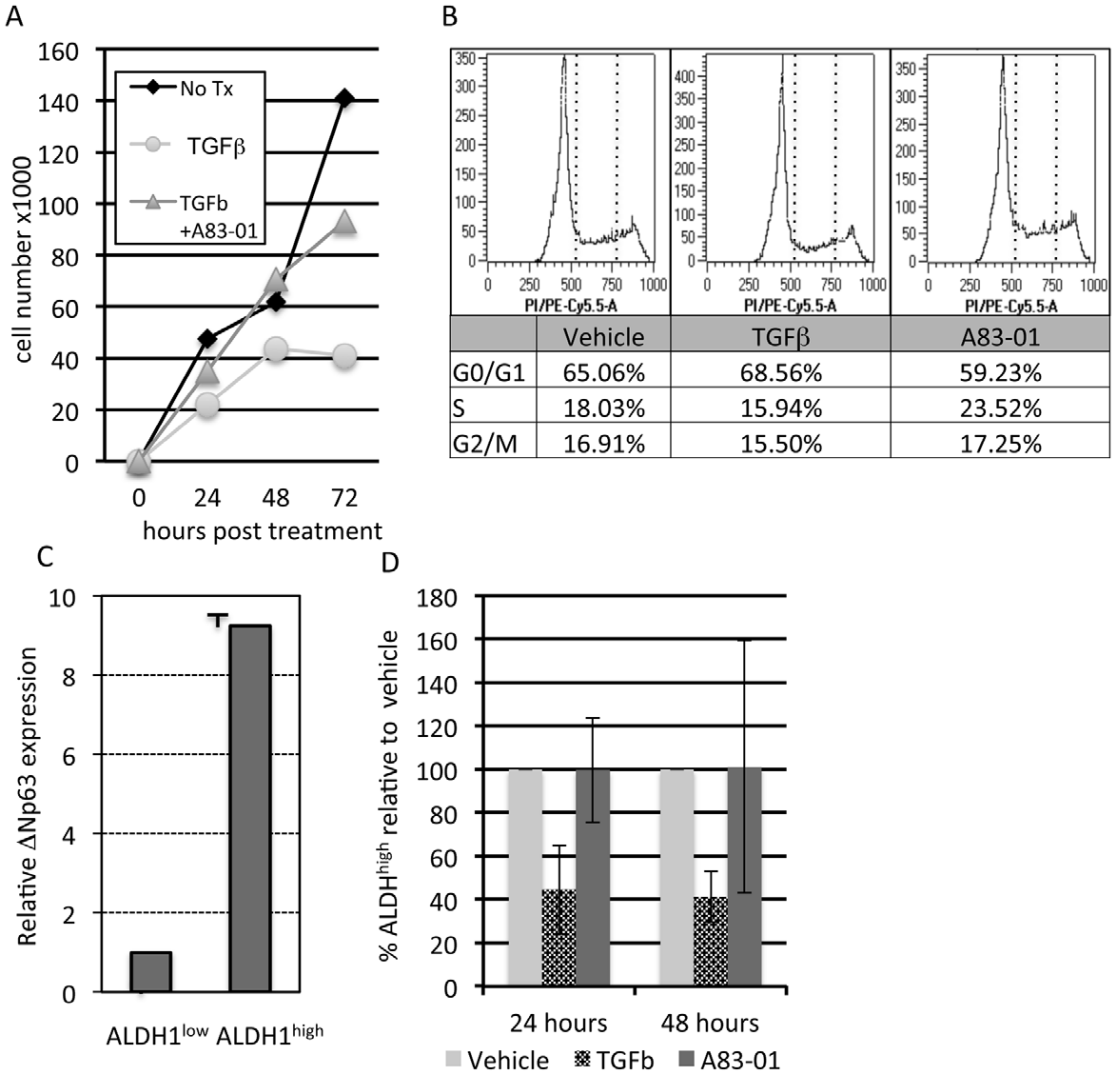
TGFΒ is anti-proliferative and suppresses ALDH1 and ΔNp63α protein levels activity in a mammary stem cell model. **A.** Cell counts plotted for IMEC cells treated with control, TGFβ1 and A83-01 for the indicated time points. Data are representative of multiple **B.** Cell cycle analysis was performed by PI staining on IMEC cells after treatment with control, TGFβ1 or A83-01 for the indicated time points. Dashed lines flank the S-phase region. **C.** IMEC cells were sorted based on the ALDH staining, ΔNp63α mRNA expression levels were analyzed using quantitative PCR. **D.** IMEC cells expressing ALDH high and low were analyzed after treating IMEC cells with vehicle control, TGFβ1 and A83-01 at the indicated time points. **E.** IMEC cells were treated with control or cycloheximide (CHX) for 2 hrs and then treated with TGFβ1 or A83-01 for 2 hrs. Whole cell lysates were analyzed for ΔNp63α and β-actin. **F.** IMEC cells were treated with control, TGFβ1 and A83-01 for 1 hr, after which cells were treated with control and CHX for 4 hrs. Whole cell lysates were analyzed for Phospho-p63 and β-actin.

The hTERT immortalized mammary epithelial cells (IMECs) were derived via retroviral transduction of the catalytic subunit of human telomerase (hTERT) into primary human mammary epithelia and clones were selected for their ability to bypass replicative senescence [32]. Subsequent analysis of multiple clonal IMEC lines indicated a basal/myoepithelial cytokeratin profile and robust expression of ΔNp63α. Other studies indicated that IMECs possess developmental potency based upon their ability to produce acinar structures with biochemically distinct basal and luminal layers [47]. Based upon these similarities to mammary stem cells, we sought to understand the biological effects of TGFΒ on IMECs and to determine the degree to which phosphorylation of ΔNp63α contributes to these effects. TGFβ caused a significant decrease in cell number over 72 hours, and this effect was reversed by co-treatment with A83-01 (Figure 5A). Cell cycle distribution analysis indicated a decrease in the population of cells in S-phase in response to TGFβ and a corresponding increase of cells in S-phase in response to A8301 (Figure 5B). To address the effects of TGFβ on stem cell activity, IMEC sub-populations with features of stem cells were enriched on the basis of high aldehyde dehydrogenase 1 (ALDH1) activity [48]. Analysis of ΔNp63α mRNA levels in ALDH1^high^ and ALDH1^low^ fractions indicated that ΔNp63α mRNA levels were significantly enriched in the ALDH1^high^ self-renewing population (Figure 5C). This enrichment for ΔNp63α expression is consistent with the assertion that ALDH1^high^ fractions of IMECs are enriched for self-renewing capacity and also indicates that TGFβ may influence this fraction via ΔNp63α phosphorylation. To test this, IMECs were treated with vehicle, TGFβ or A83-01 for 24 and 48 hours, and the ALDH1^high^ fraction was measured. Results indicated that TGFβ treatment significantly reduced ALDH1 activity in IMECs resulting in a smaller ALDH^high^ cellular fraction (Figure 5D and Figure S6) The reduction in ALDH1^high^ cells in response to TGFβ, coupled to the fact that ΔNp63α expression is increased in this fraction suggested that TGFβ might be opposing the activity of ΔNp63α. Together these data indicate that TGFβ signaling in IMECs is anti-proliferative and targets self-renewing populations that are enriched for ΔNp63α expression.

**Figure 6:**
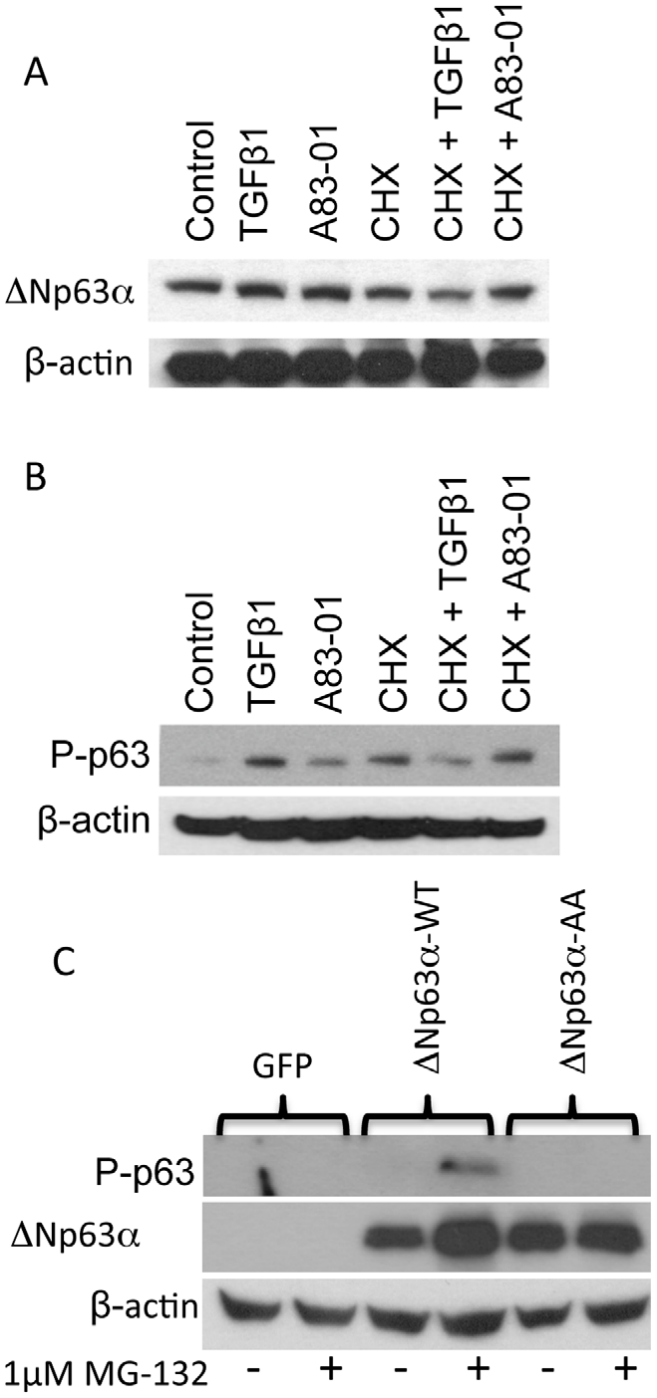
TGFΒ treatment destabilizes ΔNp63α in a manner that is dependent upon the kinase activity of ALK5. A. TGFΒ increases the rate of ΔNp63α turnover. IMECs were treated with vehicle or cycloheximide followed by treatment with vehicle, TGFΒ or A8301. B. TGFΒ-stimulation selectively increases the rate of turnover of phospho-ΔNp63α. IMECs were treated with vehicle, TGFΒ or A83-01 followed by vehicle or cycloheximide. **C.** Phospho-p63 is stabilized by the 26S proteosome inhibitor MG132. H1299 cells were transfected with GFP, ΔNp63α WT and ΔNp63α AA and treated for 2 hours with 1 µM MG-132.

The previous data suggests that TGFβ opposes the activity or expression of ΔNp63α. This coupled to previous studies indicating that phosphorylation of ΔNp63α at S66/68 leads to its destabilization suggested that TGFβ might oppose ΔNp63α is by causing its degradation. To test this, IMECs were treated with TGFβ or A83-01 in the absence or presence of cycloheximide. Under these conditions treatment of IMECs with TGFβ for 4 hours in the absence of *de novo* protein synthesis selectively repressed ΔNp63α protein levels indicating that TGFΒ may destabilize ΔNp63α (Figure 6A). To determine if the phosphorylated form of ΔNp63α was preferentially destabilized, cells were pre-treated with vehicle, TGFΒ or A83-01 for 1 hour followed by treatment with vehicle or cycloheximide for 4 hours and phospho-p63 levels were evaluated by western blot. Consistent with data in Figure 2, TGFβ stimulation leads to increased phosphorylation of ΔNp63α, however, treatment with cycloheximide resulted in the destabilization of phospho-p63 signal in the TGFβ-treated sample, indicating that TGFβ-mediated phosphorylation of ΔNp63α destabilizes ΔNp63α (Figure 6B). To determine if TGFβ-stimulated degradation of ΔNp63α was mediated by the 26 proteosome, H1299 cells were transfected with GFP, ΔNp63α-WT and ΔNp63α AA under conditions that actively promoted TGFβ signaling. Cells were then treated for 2 hours with vehicle or 1 µM MG-132. Western analysis showed that phosphorylated ΔNp63α was stabilized by MG132 indicating that phospho-ΔNp63α is degraded by the 26S proteasome. Western analysis for total ΔNp63α indicated that MG132 caused an increase in ΔNp63α-WT but not in ΔNp63α-AA, indicating that targeting of ΔNp63α to the 26S proteasome requires phosphorylation of serines 66 and 68. These findings support a model in which TGFβ-mediated phosphorylation of ΔNp63α leads to its degradation by the 26S proteasome.

### The Anti-clonogenic Effects of TGFΒ Require Phosphorylation of ΔNp63α

**Figure 7:**
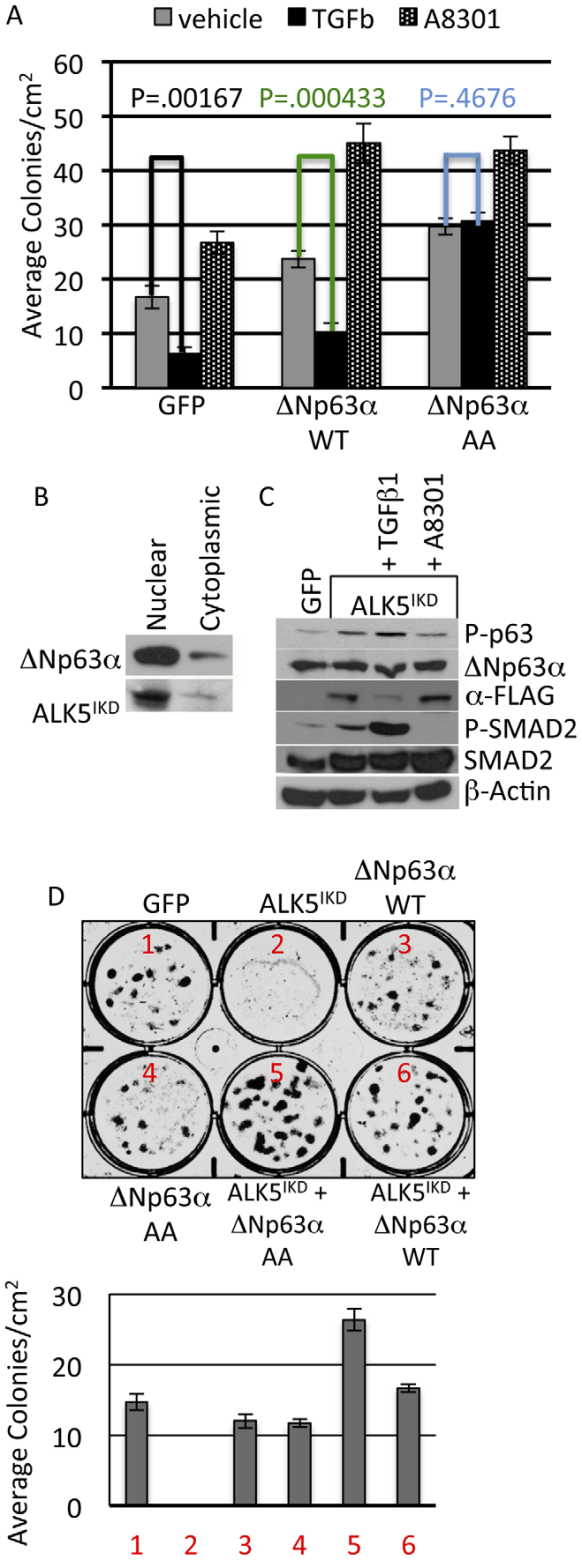
The anti-clonogenic effects of TGFΒ require phosphorylation of ΔNp63α. **A.** IMEC cells were transiently transfected with GFP, ΔNp63α-WT or ΔNp63α-AA at equimolar ratios. 48 hrs after transfection cells were subjected to G418 selection with control, TGFβ1 or A83-01. Colonies formed were then stained with crystal violet. To quantify colonies 3 randomly 1 cm^2^ spaces per well were counted and these data were used to calculate the average number of colonies per cm^2^. Error bars represent the standard deviation. A two-tailed T-Test was used to determine the significance of the effects of TGFβ on colony formation in each transfection group. **B.** ALK5^IKD^-flag expression vector was transiently transfected into IMEC cells, 24 hrs later cells were harvested for nuclear and cytoplasmic extracts. Subcellular localization of P63 and ALK5^IKD^ proteins were analyzed by immunoblotting with Anti-P63 and Anti-Flag antibodies. **C.** IMEC cells were transfected with GFP and ALK5^IKD^, 24 hrs later cells were treated with TGFβ1 and A83-01 for 1 hr and whole cell lysates were analyzed for phospho-P63, totalP63, phospho-SMAD2, total SMAD2 and β-actin via immunoblotting. **D.** IMEC cells were transfected with GFP or ALK5^IKD^ at equimolar ratios. 48 hrs after transfection cells were subjected to G418 selection until visible colonies formed which were then stained with crystal violet. **E.** IMEC cells were either transfected with GFP, ALK5^IKD^, ΔNp63α-WT and ΔNp63α-AA or ALK5^IKD^+ΔNp63α-AA and ALK5^IKD^+ΔNp63α-WT at equimolar ratios. 48 hrs after transfection cells were subjected to G418 selection. Formed colonies were then stained with crystal violet.

TGFβ signaling has been shown to be a tumor suppressive during early stages of breast cancer initiation and to promote breast cancer progression and metastasis at later stages [18]. The specific targets and signaling pathways governing these divergent effects are incompletely understood. The previous results indicate that TGFβ signaling is growth inhibitory and destabilizes ΔNp63α via phosphorylation. To determine if phosphorylation of ΔNp63α is required for the anti-proliferative effects of TGFβ, IMECs were transfected with GFP, ΔNp63α-WT or ΔNp63α-AA and selected in G418 while simultaneously being treated with either TGFβ or A83-01. Colonies were allowed to grow for 15 days and then fixed and stained with crystal violet. Consistent with the observed effects on proliferation and ALDH1 activity, TGFβ treatment was anti-clonogenic, while A83-01 promoted colony formation (Figure 7A and Figure S7A). Additionally, the anti-clonogenic effect of TGFβ was rescued by ΔNp63α -AA but not ΔNp63α -WT (Figure 7A). To determine if these differences were statistically significant, a two-tailed T-Test of the effects of TGFΒ on colony formation in each transfection group revealed that TGFβ caused a statistically significant reduction in colony formation in the GFP transfectant (P = 0.00167) and the ΔNp63α -WT transfectant (P = 0.000433), but not in the ΔNp63α-AA transfectant (P = 0.4676). This statistical analysis supports the assertion that ΔNp63α-AA was able to rescue the anti-clonogenic effects of TGFβ. The coupled to the finding that ΔNp63α-WT was unable to rescue these effects supports the conclusion that the anti-clonogenic effects of TGFβ require phosphorylation of ΔNp63α at serine 66 and 68. This result indicates that the anti-clonogenic effects of TGFβ require ΔNp63α phosphorylation. This observation coupled to data indicating that TGFβ stimulates nuclear translocation of ALK5 suggests that the intracellular kinase domain of ALK5 (ALK5^IKD^) mediates TGFβ-stimulated phosphorylation of ΔNp63α and the anti-clonogenic effects of TGFβ. To address this, an expression vector was developed to produce ALK5^IKD^ and transfected into IMECs. Consistent with the proposed model, ectopic ALK5^IKD^ accumulates in the nucleus (Figure 6B) and phosphorylates ΔNp63α in a manner that is independent of TGFΒ but sensitive to A83-01 (Figure 6C). The observation that ectopic ALK5^IKD^ is sufficient to phosphorylate ΔNp63α under TGFβ-depleted conditions is consistent with a model in which TGFβ signaling is bypassed by directly targeting ALK5^IKD^ to the nucleus. This observation predicts that ALK5^IKD^ is sufficient to recapitulate the anti-clonogenic effects of TGFβ. IMECs were transfected with expression plasmids encoding ALK5^IKD^ and either ΔNp63α-WT or ΔNp63α-AA. G418 resistant colonies were selected and quantified. Results indicated that ALK5^IKD^ was potently anti-clonogenic (Figure 6D) and that this effect was partially rescued by ΔNp63α-WT and completely rescued by ΔNp63α-AA (Figure 6E). These results confirm that the anti-clonogenic effects of TGFβ in IMECs are mediated by ALK5^IKD^ and require phosphorylation of ΔNp63α. These studies identify ΔNp63α as a novel target of TGFβ signaling and indicate that the ability of ΔNp63α to promote colony formation is potently inhibited by TGFβ.

## Discussion

We report the identification of the Type 1 TGFβ Receptor, ALK5, as a kinase that mediates phosphorylation of ΔNp63α at S66/68. Our studies indicate that TGFβ stimulation and UV irradiation also phosphorylate ΔNp63α at S66/68 and this effect is sensitive to pharmacologic inhibition of ALK5 with A83-01. We present data indicating that TGFβ is able to stimulate the nuclear translocation of ALK5 and that a 34 kDa C-terminal truncation of ALK5 preferentially translocates to the nucleus. Our studies indicate that the anti-clonogenic effects of TGFβ are mediated by ΔNp63α phosphorylation. Coupled to the established role of ΔNp63α in the long-term preservation of proliferative capacity in adult stem cells, these studies suggest that TGFβ/ALK5/ΔNp63α signaling may contribute to the proliferative capacity of adult stem cells and tumor stem cells. Together these studies describe a previously unrecognized TGFβ signaling pathway that directly impacts the proliferative capacity and clonogenicity of ΔNp63α-positive cells. Additional studies will be necessary to determine the degree to which this pathway accounts for the effects of TGFβ on the activity of adult stem cells. Previous studies have shown that TGFβ promotes oncogene-induced senescence (OIS) in a manner that is independent of p53 [49]. Separately ΔNp63α has been shown to be a potent suppressor of OIS [13]. Here we present data indicating that TGFβ activation destabilizes ΔNp63α a suggesting a potential mechanism by which TGFβ promotes OIS. This report establishes a novel signaling relationship between TGFβ and TP63 and demonstrates that this relationship underlies aspects of adult stem cell biology that are governed by ΔNp63α. Finally it will be important to elucidate the role of this signaling pathway in Epithelial to Mesenchymal Transition. Recent studies indicate that ΔNp63α opposes EMT [50]–[51] suggesting that TGFβ-mediated destabilization of ΔNp63α may be an important step in EMT, a process that is critical for cancer progression and metastasis. Additional studies will be necessary to systematically evaluate the cellular consequences of TGFβ-mediated phosphorylation of ΔNp63α.

Previous studies have shown that ΔNp63α is phosphorylated at S66/68 in response to UV irradiation [29]. The observation that UV-initiated phosphorylation of ΔNp63α was sensitive to A83-01 implicates ALK5 in the cellular response to stress, however, additional studies will be necessary to determine if ALK5-mediated ΔNp63α phosphorylation contributes to the role of TGFβ signaling in promoting metastasis following ionizing radiation [41]. Similarly, it will be of significant interest to determine if the potent TGFβ response of the tumor microenvironment to radiation [42] contributes to ΔNp63α phosphorylation. Coupled to the finding that TGFβ-mediated phosphorylation of ΔNp63α is anti-proliferative and anti-clonogenic, the ALK5-mediated response to cellular stress may act as a protective mechanism that limits proliferation under conditions of cellular or genotoxic stress. Additional studies will be necessary to determine if the mechanisms underlying activation of ALK5 in response to cellular stress are TGFβ-dependent or TGFβ-independent. Furthermore, it will be potentially clinically relevant to determine the biological consequences of ALK5-mediated phosphorylation of ΔNp63α in enriched tumor stem cell fractions. Finally, ΔNp63α has been shown to act as a survival factor that mediates therapeutic resistance and opposes apoptosis in breast cancers with a basal phenotype [15] and also in squamous cell carcinomas of the head and neck [16]. It will be of interest to determine if disruption of the TGFβ/ALK5/ΔNp63α signaling pathway subverts these activities thereby overcoming therapeutic resistance.

Finally, recent studies have indicated unacceptable levels of cardiac and inflammatory toxicity associated with selective ALK5 kinase inhibitors and it is likely that these adverse effects will limit their development and clinical utility [52]. Given the remarkably pleiotropic actions of TGFβ, it is not surprising that drugs that disrupt all ALK5 signaling would have a wide range of effects. This highlights the need to identify specific signaling pathways downstream of TGFβ that account for specific activities of TGFβ. Doing so will make it possible to target specific actions of TGFβ while avoiding adverse side effects. Data presented here support a model in which unknown proteolytic activity mediates the translocation of ALK5^IKD^. This implies that inhibition of this protease may result in disruption of ΔNp63α phosphorylation. Further studies will be necessary to identify this protease, however a recent study has shown that the TNF-α Converting Enzyme (TACE) is able to mediate proteolysis of ALK5 and that TACE activity is required for accumulation of ALK5 in the nucleus [53]. The specific relevance of this finding to the generation of ALK5^IKD^ and phosphorylation of ΔNp63α is unknown because in that study TACE was shown to target the ALK5 ectodomain, which is predicted to produce a fragment greater than 34 kDa. This also raises questions regarding the mechanism(s) by which an ALK5 fragment that retains the transmembrane domain might translocate to the nucleus. Presently the protease(s) that account for generation of ALK5^IKD^ remain unknown and their identification represents an important step in testing our model for ALK5-mediated ΔNp63α phosphorylation and also in identifying pharmacologically accessible pathway components.

## Supporting Information

Figure S1 Schematic representation of the siRNA-based screen of the human kinome. The table at the bottom lists the top 14 hits in the screen showing the data produced from fluorescence plate readings of the IF and subsequent Abs_600_ readings for crystal violet staining. Primary hits progressed to the secondary screen and kinases for which all three siRNAs repressed phosphorylation of DNp63a were selected.
(PDF)

Figure S2 The molecular determinants of DNp63a phosphorylation by ALK5 are distinct from those necessary for SMAD2/3 phosphorylation. H1299 cells were co-transfected with wild-type ALK5, the T202D mutant which had previously been shown to constitutively activate TGFb signaling, the K232R mutant which had been previously shown to inhibit TGFb signaling and an ALK5-GFP fusion. At 24 hours post transfection protein was harvested and analyzed by western blot. Comparison of the P-p63 and P-SMAD2/3 signals indicated that T202D was unable to phosphorylate DNp63a but was able to phosphorylate SMAD2/3 (Lane 3). Remarkably the K232R mutant was able to phosphorylate DNp63a but not SMAD2/3 (Lane 4). These results suggest that the molecular mechanisms by which ALK5 phosphorylates DNp63a are distinct from those that phosphorylate SMAD2/3.
(PDF)

Figure S3 Effects of three TGFbR2-directed siRNAs on expression of TGFbR2 and SMAD2 phosphorylation. H1299 cells were transfected with the indicated siRNAs and TGFbR2 and phospho-SMAD2 were analyzed to confirm the efficacy of the siRNA. SiRNA-C was used in the experiment shown in Figure 2C.
(PDF)

Figure S4 Schematic representation of signal transduction pathways known to be downstream of the TGFb receptor complex. Kinases associated with these pathways are shown in pink and the phospho-p63 vs total p63 IF score is shown as is the relationship of that score to the mean.
(PDF)

Figure S5 Transfection of H1299 cells with ALK5-directed siRNA ablates immunoflourescent detection of ALK5. This data confirms the specificity of ALK5 detection presented in Figure 4E. This data confirms the selectivity of the ALK5 antibody.
(PDF)

Figure S6 Repesentative Aldefluor data from which Figure 5D was derived. Negative controls using the ALDH1 inhibitor DEAB are used to establish the gate separating ALDH^Low^ from ALDH^High^ fractions.
(PDF)

Figure S7 The anti-clonogenic effects of TGFb are phenocopied by ectopic ALK5IKD. **A.** The anticlonogenic effects of TGFb on IMECs are partially rescued by the phospho-ablative DNp63a-AA mutant. Colony forming assay shown is representative of multiple experiments and corresponds to the graphical data displayed in Figure 7A. **B.** Ectopic expression of ALK5^IKD^ is anti-clonogenic in IMEC cells. IMECs were tranfected with pcDNA3.1-GFP and pcDNA3.1-ALK5^IKD^ and selected in 200 µg/ml G418 for 12 days. Colonies were fixed in alcohol and stained with crystal violet. Graph at right represents a quantification of the colony formation in which colonies from three random 1 cm × 1 cm squares were analyzed using ImageJ software. Bars represent the average of three counts and error bars represent the standard error of the mean.
(PDF)
