# Supplementary material for: Phosphorylation of ΔNp63α via a Novel TGFβ/ALK5 Signaling Mechanism Mediates the Anti-Clonogenic Effects of TGFβ
Source: PLoS One. 2012 Nov 16;7(11):e50066. doi: 10.1371/journal.pone.0050066 (PMC3500343; doi:10.1371/journal.pone.0050066)
Supplement: Figure S1 — Schematic representation of the siRNA-based screen of the human kinome. The table at the bottom lists the top 14 hits in the screen showing the data produced from fluorescence plate readings of the IF and subsequent Abs600 readings for crystal violet staining. Primary hits progressed to the secondary screen and kinases for which all three siRNAs repressed phosphorylation of DNp63a were selected. (PDF) [file pone.0050066.s001.pdf]

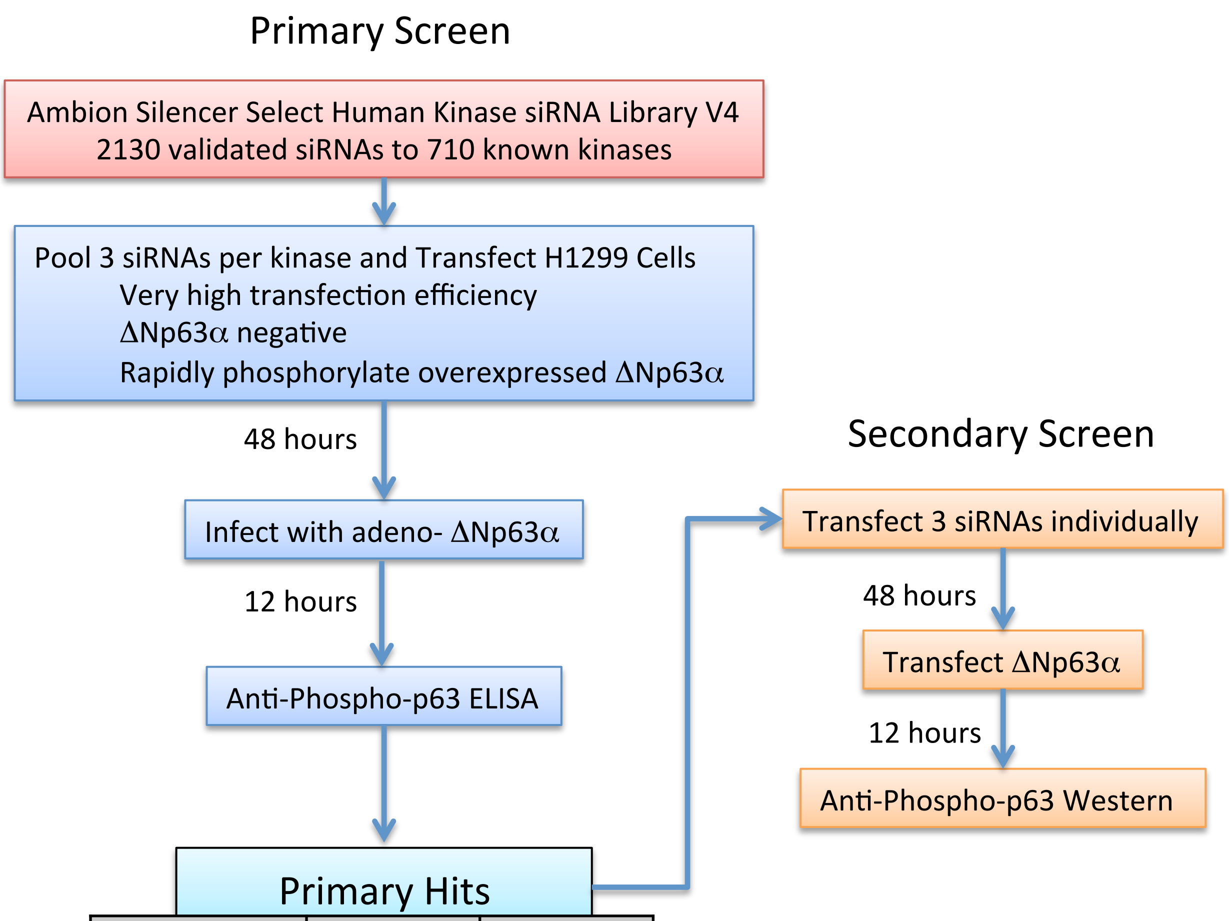

Gene Symbol	Raw Kinase Data	C.V. Data
PCTK3	2.598	0.127
PIK3CD	2.698	0.149
FRK	2.983	0.127
GALK1	2.984	0.125
TGFBR1	3.039	0.143
CKB	3.053	0.129
PHKG1	3.115	0.134
SH3BP5L	3.115	0.136
NTRK2	3.133	0.13
PRKAA2	3.149	0.132
PLK3	3.197	0.121
GRK6	3.213	0.157
PRPS1L1	3.218	0.14
BTK	3.236	0.13

Figure S1: Schematic representation of the siRNA-based screen of the human kinome. The table at the bottom lists the top 14 hits in the screen showing the data produced from fluorescence plate readings of the IF and subsequent Abs_{600} readings for crystal violet staining. Primary hits progressed to the secondary screen and kinases for which all three siRNAs repressed phosphorylation of ΔNp63α were selected.

Cherukuri et al Figure S1
