# Supplementary material for: Phosphorylation of ΔNp63α via a Novel TGFβ/ALK5 Signaling Mechanism Mediates the Anti-Clonogenic Effects of TGFβ
Source: PLoS One. 2012 Nov 16;7(11):e50066. doi: 10.1371/journal.pone.0050066 (PMC3500343; doi:10.1371/journal.pone.0050066)
Supplement: Figure S2 — The molecular determinants of DNp63a phosphorylation by ALK5 are distinct from those necessary for SMAD2/3 phosphorylation. H1299 cells were co-transfected with wild-type ALK5, the T202D mutant which had previously been shown to constitutively activate TGFb signaling, the K232R mutant which had been previously shown to inhibit TGFb signaling and an ALK5-GFP fusion. At 24 hours post transfection protein was harvested and analyzed by western blot. Comparison of the P-p63 and P-SMAD2/3 signals indicated that T202D was unable to phosphorylate DNp63a but was able to phosphorylate SMAD2/3 (Lane 3). Remarkably the K232R mutant was able to phosphorylate DNp63a but not SMAD2/3 (Lane 4). These results suggest that the molecular mechanisms by which ALK5 phosphorylates DNp63a are distinct from those that phosphorylate SMAD2/3. (PDF) [file pone.0050066.s002.pdf]

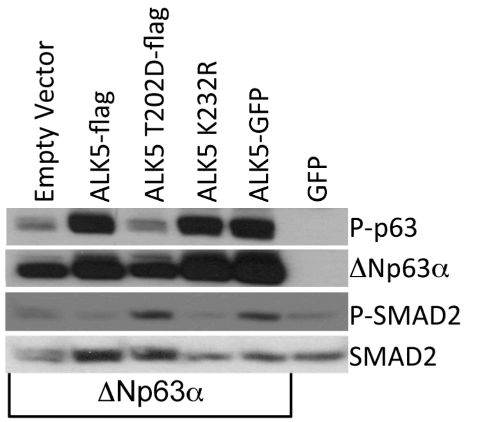

Figure S2: The molecular determinants of ΔNp63α phosphorylation by ALK5 are distinct from those necessary for SMAD2/3 phosphorylation. H1299 cells were co-transfected with wild-type ALK5, the T202D mutant which had previously been shown to constitutively activate TGFb signaling, the K232R mutant which had been previously shown to inhibit TGFb signaling and an ALK5-GFP fusion. At 24 hours post transfection protein was harvested and analyzed by western blot. Comparison of the P-p63 and P-SMAD2/3 signals indicated that T202D was unable to phosphorylate ΔNp63α but was able to phosphorylate SMAD2/3 (Lane 3). Remarkably the K232R mutant was able to phosphorylate ΔNp63α but not SMAD2/3 (Lane 4). These results suggest that the molecular mechanisms by which ALK5 phosphorylates ΔNp63α are distinct from those that phosphorylate SMAD2/3.

Cherukuri et al Figure S2
