# Supplementary material for: Phosphorylation of ΔNp63α via a Novel TGFβ/ALK5 Signaling Mechanism Mediates the Anti-Clonogenic Effects of TGFβ
Source: PLoS One. 2012 Nov 16;7(11):e50066. doi: 10.1371/journal.pone.0050066 (PMC3500343; doi:10.1371/journal.pone.0050066)
Supplement: Figure S3 — Effects of three TGFbR2-directed siRNAs on expression of TGFbR2 and SMAD2 phosphorylation. H1299 cells were transfected with the indicated siRNAs and TGFbR2 and phospho-SMAD2 were analyzed to confirm the efficacy of the siRNA. SiRNA-C was used in the experiment shown in Figure 2C. (PDF) [file pone.0050066.s003.pdf]

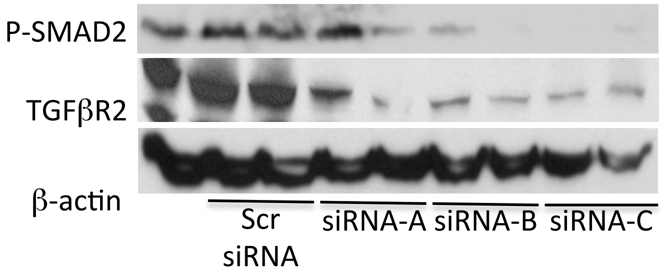

Figure S3: Effects of three TGFβR2-directed siRNAs on expression of TGFβR2 and SMAD2 phosphorylation. H1299 cells were transfected with the indicated siRNAs and TGFβR2 and phospho-SMAD2 were analyzed to confirm the efficacy of the siRNA. SiRNA-C was used in the experiment shown in Figure 2C.
