# Supplementary material for: Phosphorylation of ΔNp63α via a Novel TGFβ/ALK5 Signaling Mechanism Mediates the Anti-Clonogenic Effects of TGFβ
Source: PLoS One. 2012 Nov 16;7(11):e50066. doi: 10.1371/journal.pone.0050066 (PMC3500343; doi:10.1371/journal.pone.0050066)
Supplement: Figure S4 — Schematic representation of signal transduction pathways known to be downstream of the TGFb receptor complex. Kinases associated with these pathways are shown in pink and the phospho-p63 vs total p63 IF score is shown as is the relationship of that score to the mean. (PDF) [file pone.0050066.s004.pdf]

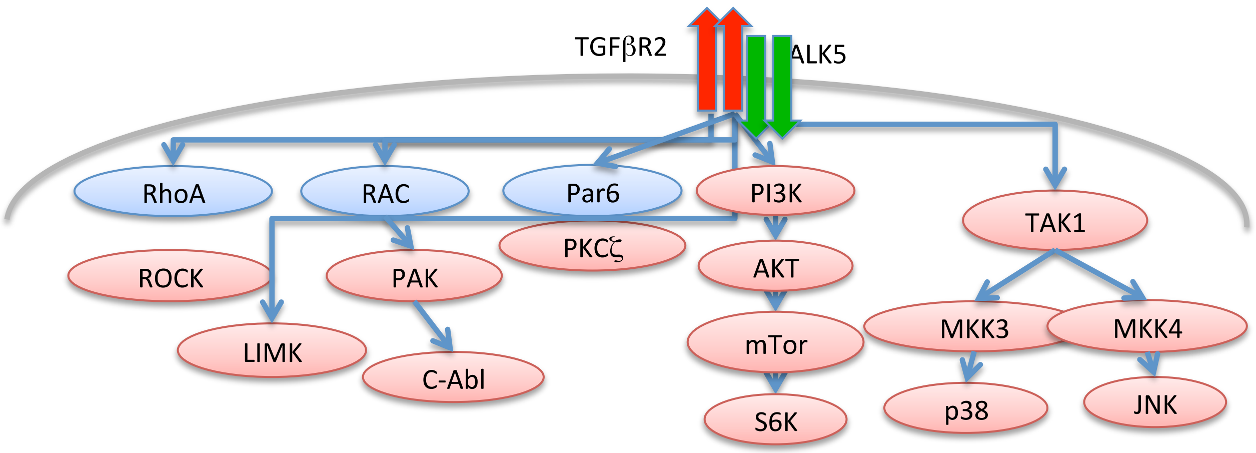

Kinase	Score	StDev from Mean
ALK5	3.039	-2.252
AKT1	3.419	-1.734
AKT2	3.488	-1.640
Abl	3.753	-1.278
MKK3	5.52	-1.132
p70S6K	4.088	-0.821
TAK1	4.225	-0.634
PKCz	4.579	-0.151
LIMK	4.739	0.067
PI3K	4.755	0.089
AKT3	4.983	0.400
MKK4	5.095	0.553
ROCK	5.43	1.010
PAK	5.799	1.513
mToR	6.152	1.995

Figure S4: Schematic representation of signal transduction pathways known to be downstream of the TGFβ receptor complex. Kinases associated with these pathways are shown in pink and the phospho-p63 vs total p63 IF score is shown as is the relationship of that score to the mean.
