# Supplementary material for: Phosphorylation of ΔNp63α via a Novel TGFβ/ALK5 Signaling Mechanism Mediates the Anti-Clonogenic Effects of TGFβ
Source: PLoS One. 2012 Nov 16;7(11):e50066. doi: 10.1371/journal.pone.0050066 (PMC3500343; doi:10.1371/journal.pone.0050066)
Supplement: Figure S5 — Transfection of H1299 cells with ALK5-directed siRNA ablates immunoflourescent detection of ALK5. This data confirms the specificity of ALK5 detection presented in Figure 4E. This data confirms the selectivity of the ALK5 antibody. (PDF) [file pone.0050066.s005.pdf]

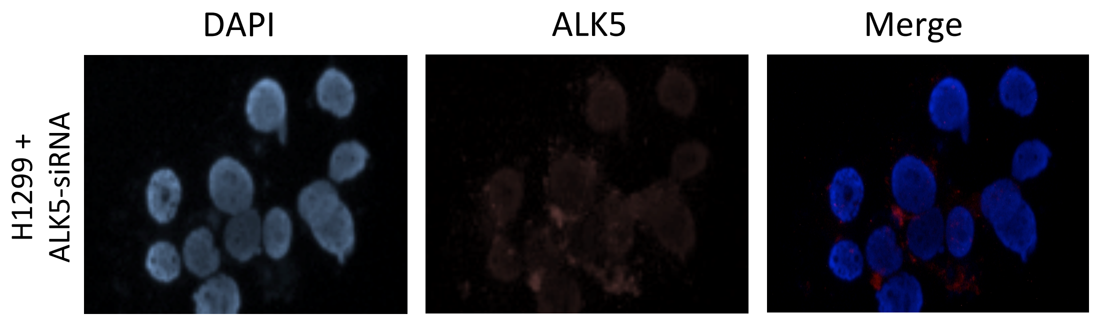

Figure S5: Transfection of H1299 cells with ALK5-directed siRNA ablates immunoflourescent detection of ALK5. This data confirms the specificity of ALK5 detection presented in Figure 4E. This data confirms the selectivity of the ALK5 antibody.
