# Supplementary material for: Phosphorylation of ΔNp63α via a Novel TGFβ/ALK5 Signaling Mechanism Mediates the Anti-Clonogenic Effects of TGFβ
Source: PLoS One. 2012 Nov 16;7(11):e50066. doi: 10.1371/journal.pone.0050066 (PMC3500343; doi:10.1371/journal.pone.0050066)
Supplement: Figure S6 — Repesentative Aldefluor data from which Figure 5D was derived. Negative controls using the ALDH1 inhibitor DEAB are used to establish the gate separating ALDHLow from ALDHHigh fractions. (PDF) [file pone.0050066.s006.pdf]

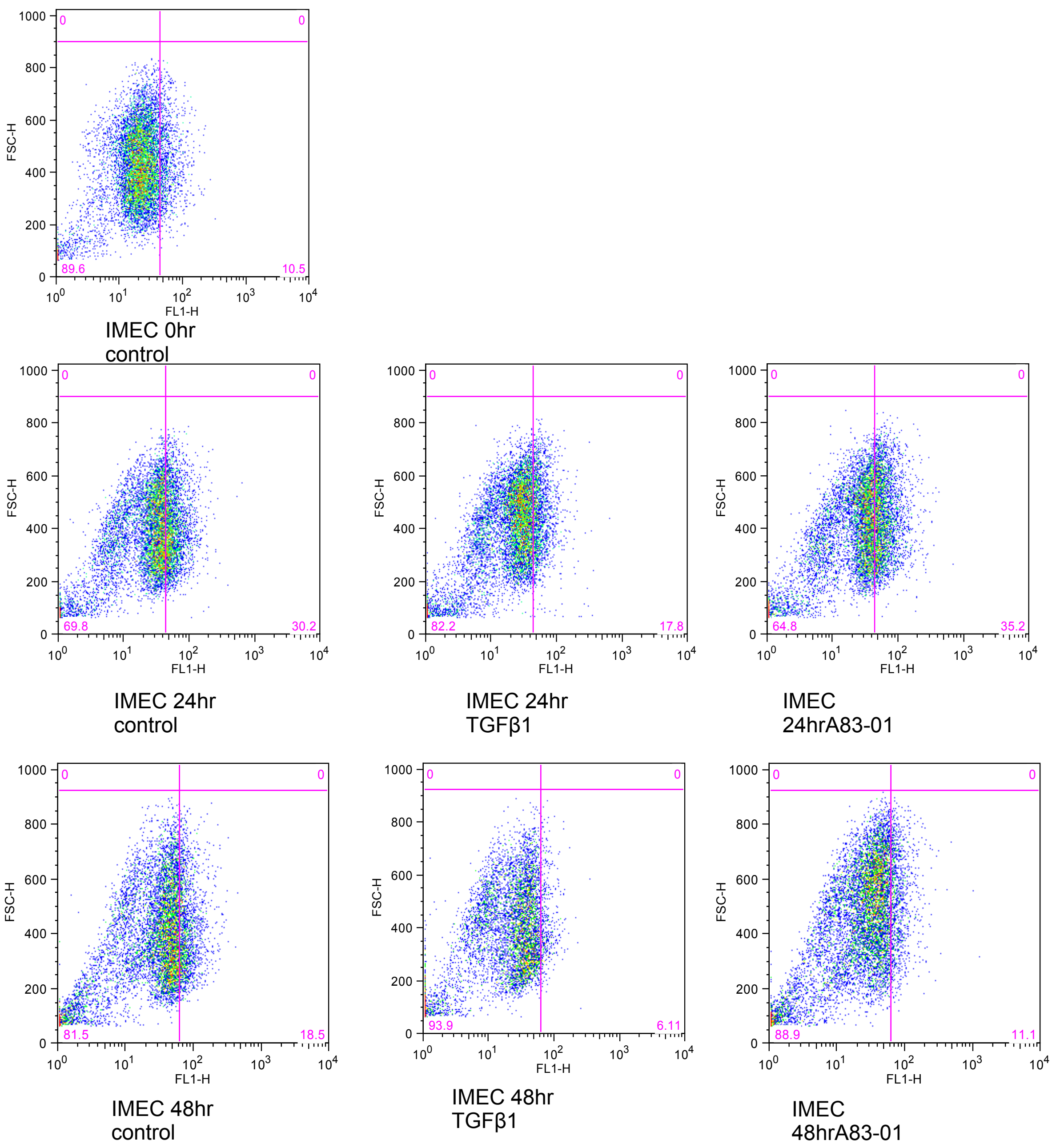

Figure S6: Repesentative Aldefluor data from which Figure 5D was derived. Negative controls using the ALDH1 inhibitor DEAB are used to establish the gate separating ALDH^{Low} from ALDH^{High} fractions.
