# Supplementary material for: Phosphorylation of ΔNp63α via a Novel TGFβ/ALK5 Signaling Mechanism Mediates the Anti-Clonogenic Effects of TGFβ
Source: PLoS One. 2012 Nov 16;7(11):e50066. doi: 10.1371/journal.pone.0050066 (PMC3500343; doi:10.1371/journal.pone.0050066)
Supplement: Figure S7 — The anti-clonogenic effects of TGFb are phenocopied by ectopic ALK5IKD. A. The anticlonogenic effects of TGFb on IMECs are partially rescued by the phospho-ablative DNp63a-AA mutant. Colony forming assay shown is representative of multiple experiments and corresponds to the graphical data displayed in Figure 7A. B. Ectopic expression of ALK5IKD is anti-clonogenic in IMEC cells. IMECs were tranfected with pcDNA3.1-GFP and pcDNA3.1-ALK5IKD and selected in 200 µg/ml G418 for 12 days. Colonies were fixed in alcohol and stained with crystal violet. Graph at right represents a quantification of the colony formation in which colonies from three random 1 cm × 1 cm squares were analyzed using ImageJ software. Bars represent the average of three counts and error bars represent the standard error of the mean. (PDF) [file pone.0050066.s007.pdf]

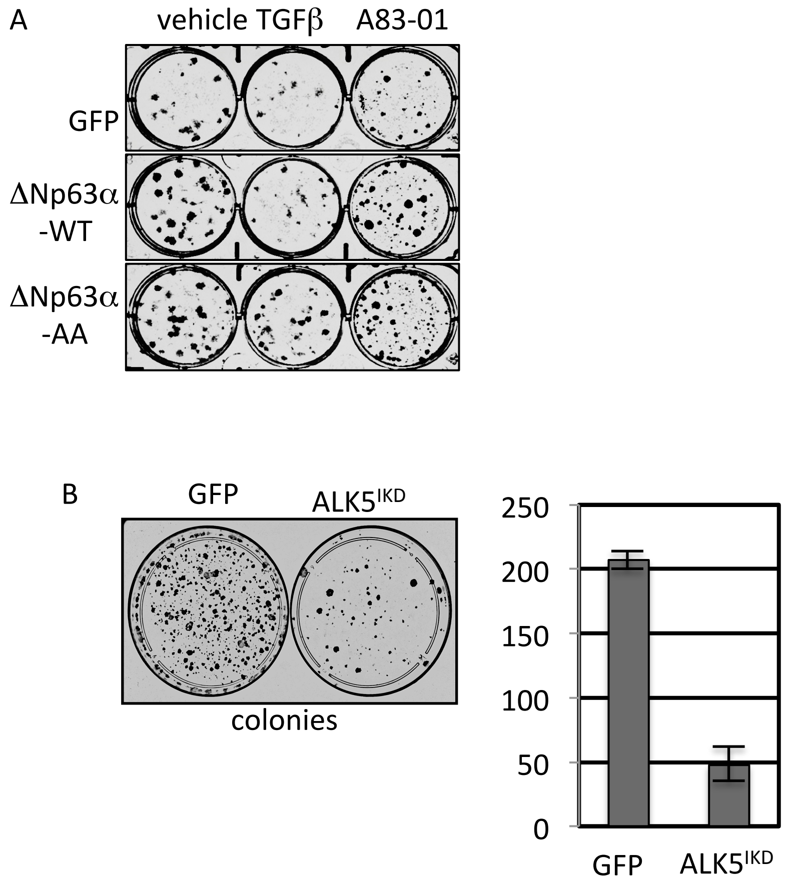

Figure S7: The anti-clonogenic effects of TGFb are phenocopied by ectopic ALK5IKD. A. The anticlonogenic effects of TGFb on IMECs are partially rescued by the phospho-ablative DNp63a-AA mutant. Colony forming assay shown is representative of multiple experiments and corresponds to the graphical data displayed in Figure 7A. **B.** Ectopic expression of ALK5^{IKD} is anti-clonogenic in IMEC cells. IMECs were tranfected with pcDNA3.1-GFP and pcDNA3.1-ALK5^{IKD} and selected in 200 µg/ml G418 for 12 days. Colonies were fixed in alcohol and stained with crystal violet. Graph at right represents a quantification of the colony formation in which colonies from three random 1 cm x 1cm squares were analyzed using ImageJ software. Bars represent the average of three counts and error bars represent the standard error of the mean.
